# The ethics of nursing care for transgender people

**DOI:** 10.1590/0034-7167-2022-0797

**Published:** 2023-12-04

**Authors:** Enrique Oltra-Rodríguez, Eva González-López, Sofía Osorio-Álvarez, Andrea Rodríguez-Alonso

**Affiliations:** I Facultad de Enfermería de Gijón. Gijón, Asturias, Spain; II Facultad de Enfermería de Gijón. Hospital de Cruz Roja de Gijón. Gijón, Asturias, Spain

**Keywords:** Ethics, Transgender Persons, Nursing Care, Sex Differentiation, Social Construction of Gender, Ética, Pessoas Transgênero, Cuidados de Enfermagem, Diferenciação Sexual, Construção Social do Gênero, Ética, Personas Transgénero, Atención de Enfermería, Diferenciación Sexual, Construcción Social del Género

## Abstract

**Objectives:**

to discuss ethical aspects in nursing care for transgender people.

**Methods:**

reflective study based on the dilemmas that emerges in nursing care for transgender people. The report was structured around the four bioethical principles.

**Results:**

health care for trans people is complex, transversal to many devices and specialties and longitudinal in time, that is why it requires coordinated action. There is an ethical framework in which the nursing care must be observed in the care of this group.

**Final Considerations:**

the nurse as a health worker can assume several general lines in the care of transgender patients. So, complementary training should be provided not only to professionals, but also to students of nursing and other health sciences.

## RESUMEN

**Objetivos:**

debatir sobre aspectos éticos en la atención de enfermería a personas transgénero.

**Métodos:**

estudio reflexivo fundamentado sobre los dilemas que se plantean en los cuidados de enfermería a personas transgénero. El relato se ha estructurado en torno a los cuatro principios bioéticos.

**Resultados:**

la atención sanitaria a las personas trans es compleja, transversal a muchos dispositivos y especialidades y longitudinal en el tiempo por lo que precisa de la actuación coordinada. Existe un marco ético en el que se encuadran los cuidados de enfermería que se precisan en la atención a este colectivo.

**Consideraciones Finales:**

la enfermera como agente de salud puede asumir diversas líneas generales en la atención a pacientes transgénero. Para ello, se debe brindar formación adicional no solo a los profesionales, también a los estudiantes de enfermería y de las demás ciencias de la salud.

## RESUMO

**Objetivos:**

discutir aspectos éticos na assistência de enfermagem às pessoas transgênero.

**Métodos:**

estudo reflexivo a partir dos dilemas que surgem no cuidado de enfermagem às pessoas transgênero. O relato foi estruturado em torno dos quatro princípios bioéticos.

**Resultados:**

a atenção à saúde de pessoas trans é complexa, transversal a muitos dispositivos e especialidades e longitudinal no tempo, por isso requer ação coordenada. Existe um referencial ético no qual se enquadram os cuidados de enfermagem que devem ser observados no atendimento a esse grupo.

**Considerações Finais:**

o enfermeiro como agente de saúde pode assumir diversas linhas gerais no atendimento a pacientes transgênero. Para tal, deve ser proporcionada formação complementar não só aos profissionais, mas também aos estudantes de enfermagem e outras ciências da saúde.

## INTRODUCTION

In the human species, the genetic determination of gender is chromosomal, homogametic for the female and heterogametic for the male, which conditions gonadal development, with some possible and infrequent variations. In a complex process mediated by the consecutive secretion of gender hormones (fundamentally androgens) in a proportional, meticulous and sensitive way in each gender and at each moment of prenatal development, secondary differentiations are produced. This impregnation and sexual differentiation affect all cells, tissues and organs, both structurally and biochemically. It is very important to note that the brain is also affected in the sexual process, and although at the beginning of the 20th century the endocrinologist and sexologist Dr. Gregorio Marañón said that “The brain is the most important sexual organ of the human being”, it is only very recently, mainly due to the contributions of neuroimaging and genetic analysis techniques, among others, that solid knowledge has been acquired about the sexuation process and more specifically on brain sexuation and, consequently, on the perception of differences in sexual identity. As with gonadal differentiation, in the internal and external genitalia, in the hormonal characteristics and even secondary sexual characteristics, there are cases that deviate from the majority process or statistical normality, it also seems that brain differentiation occurs, and this has consequences for self-perception of identity, which may differ from the biological gender^(1)^.

One of the results of the whole process of sexual differentiation is the acquisition of sexual identity, ego sexuation, that is, how everyone perceives themselves as a man or a woman. For most people, ego sexuation coincides with the sex assigned or designated by their environment at birth, allosexuality, and which is based fundamentally on the observation of the external genitalia. After this initial sexing, the whole gender construction process begins, male or female, starting with the attribution of the name, treatment in creation and education, etc.^(2)^.

If the two perceptions, ego sexuation and allosexuation, coincided, it would be a situation of cissexuality or cisgender, while if they did not coincide, it would be transsexuality or transgender^(2)^. From here, the concept “trans” will be used, since it is quite complex and goes beyond the objectives of this article - to explain the difference between the concepts of transsexuality and transgender.

Both in mythology and in classical cultures, as well as in various ethnic groups and current cultures, sexual identities emerged that could be similar to those that in our society today is known as transsexuals, people who do not accept their biological gender or their assigned gender, or even those who do not fit into the male/female dichotomous categories. These people are known in various fields as the third gender: *hijra* in Pakistan, *khanith* in Oman, *fa’afafine* in Samoa, *muxes* in Mexico...; they are generally not considered a problem and are even seen socially as a positive value.

It is not easy to know the magnitude of the trans phenomenon because studies are few and those that exist are partial and generally cannot be compared because they are based on different registers, definitions and methodologies. According to a meta-analysis carried out in 2015, based on 250 studies carried out in 9 countries, the overall prevalence of transsexuality was 4.6 in 100000 people: 6.8 for trans women and 2.6 for trans men^(3)^. Over the past 50 years, there has been an increase in prevalence. These data are related to people who resort to health systems and, therefore, it can be expected that the social reality is greater, although the value is unknown. This increase in prevalence that seems to be accelerating in recent times raises a debate about its interpretation, some argue that, due to the current greater social permissiveness, cases arise that some time ago did not dare, while other authors are very critical and attribute to other factors such as personal dissatisfactions, personality crises, typical of adolescence, and even a certain social fashion induced by the Queer movement^(4)^.

There are two aspects in which a high level of consensus is observed. The first one is that trans people tend to suffer from a worse quality of life, physical and mental health than the general population, related to the situation of vulnerability they suffer. The second one is that they often maintain a relationship with the health systems, either because of health problems or because of the endocrinological and surgical techniques that health care offers them to adjust their body appearance to the perceived gender pattern and the corresponding gender^(5-6)^.

Currents of thought are also emerging within the trans movement that disagree with the general essentialist approach and that question the relevance of having to redesign or transform the body of those who present incongruence with the perception of their gender, attributing this incongruence to the social sclerosis of the concepts of sex-gender^(7)^.

The health needs of transgender people and the care that health systems and professionals provide or deny them mean that several dilemmas are constantly raised and open up debates ranging from ideological to operational and even legislative, and those debates are not alien to nursing as a care profession, accompaniment and defense of people who suffer or need help.

As a guideline for the professional positioning of nurses, an attempt will be made to ethically analyze some of the dilemmas that arise when caring for transgender people, using the model of four principles established by Beauchamp and Childress in Principles of Biomedical Ethics^(8)^, which was later qualified by Professor Diego Gracia, hierarchizing these principles^(9)^.

## OBJECTIVES

To discuss ethical aspects in nursing care for transgender people.

## METHODS

Reflective study in which the most common dilemmas encountered when carrying out a narrative bibliographic review on nursing care for transgender people are analyzed. The analysis will be carried out around the four bioethical principles that constitute the so-called “Hierarchical Principle-ism”, which are: the two principles of minimum requirement that must be fulfilled in all actions to be considered ethical; non-maleficence and justice; while the two principles considered as maximum or of bioethical excellence are: beneficence and autonomy.

## RESULTS

As indicated in article 1.2 of the Code of Ethics for Nurses of the International Council of Nursing (ICN) (2021): “Nurses promote an environment in which everyone recognizes and respects the human rights, values, customs, religious and spiritual beliefs of the person, families and communities”^(10)^. It is within this ethical framework that the nursing care required in the care of transgender people will be framed.

### Non-maleficence

This principle, essential for any action to be considered ethical, stems from the Latin aphorism *primum non nocere* (first do no harm). In the care of trans people, it has a transcendent application both at the care level and at the community preventive level.

Care must be oriented to avoid the damage that any type of discrimination can cause and that can range from stereotyping these people when they declare themselves trans, making assumptions about their sexual practices or ways of life, to derogatory reactions or disrespect to the name or pronoun with which they are identified. Likewise, sanitary facilities must be adapted to ensure the necessary privacy and dignified treatment.

Transgender people, who so need it, should receive the most appropriate and safe hormone treatments and forms of administration, as indicated by the World Health Organization in its Guidelines on self-care interventions for health and well-being (2022 revision).

No less important are the community health education interventions in schools and families aimed at accepting sexual diversity and, within it, the underage trans. Acceptance is the first necessary step to protect and avoid situations of marginalization or mistreatment, which are the direct cause of the so-called gender dysphoria. Transcendent for the acceptance is the early detection of underage trans within the family and that they understand the nature of the phenomenon so that they can deal with it in a positive way.

### Justice

This principle is based on providing more care to those who need it the most. The health inequities of this group are recognized, related to the laws and rights recognized in different countries. These inequities occur both in those who start the transition process and in the collective in general, considering the health conditions to which they are forced on multiple occasions, such as socioeconomic and labor situations, mental health and marginality.

It should be part of nurses’ advocacy to encourage public health initiatives involving transgender people, ensure the competence of health professionals for this group and monitor compliance with non-discriminatory policies both in health systems and in society in general.

When carrying out an economic assessment of the transition process, not only the direct costs of health care, basically surgical and hormonal, should be considered, but a rigorous Assessment of Health Technologies should be carried out in which direct, indirect, health and non-sanitary costs are considered, as well as those of difficult tangibility.

### Beneficence

As health professionals and if they intend to provide excellent care, they have an ethical obligation to do good if it does not imply a risk to their way of life or coexistence.

Both the process of adaptation and acceptance by families in the case of underage people, and the process of transition or gender reassignment in adults involve a complex and sometimes tortuous and labyrinthine journey.

It is beneficial to monitor and advise families in the community, promoting support and self-help services and networks, in addition to bringing the population closer to knowledge of the trans phenomenon based on science so that citizens can understand it and, consequently, accept and respect it.

People who decide to go through a process of harmonizing their sexual characteristics must go through an intricate journey through the different services of the health systems, simplify it and implement strategies aimed at the specific health care of this population and offer follow-up through figures as a case manager nurse or similar also supposes a beneficial action.

### Autonomy

This principle of bioethical excellence is also transcendent, it means facilitating people to make decisions according to their way of being in the world, it implies allowing the right to recognition of the trans condition that is neither volitional, nor apprehensible, nor teachable, nor ephemeral, nor capricious. Therefore, conditions and support are necessary, but also understandable and true information, especially when decisions will condition actions that are difficult or impossible to reverse.

Nurses who capture situations of need, in their role as advocates for patients, should have the responsibility of putting those who need it in contact with specialized professionals or with solvent organizations that provide this information. According to article 1.3 of the ICN code of ethics^(10)^, “nurses ensure that the person and the family receive understandable, accurate, sufficient and timely information, in a way that is appropriate to the cultural, linguistic, cognitive and physical needs of the patient, in addition to their psychological state, on which to base their consent to care and corresponding treatment”.

In the case of underage people, although the perception of sexuality corresponds to the individual, autonomy in decision-making corresponds to their legal representatives. It will be necessary to guide the family unit towards early detection, follow up by providing references, contribute to the authentication of the story, rule out interference, respect the process and even accept evanescence situations, if they occur, and encourage prudence, delaying irreversible interventions as much as possible, that is, to provide underage people with as much time as possible for personal maturation so that they can experience and establish the relationship they deem most appropriate with their sexual identity, their body and their gender role, protecting them and always avoiding situations of suffering or marginalization.

## FINAL CONSIDERATIONS

Currently, the increase in the prevalence of trans people is worrying, contemplating several hypotheses and requiring more consistent studies. Therefore, especially in childhood and adolescence, the processes must be respected, but acting with prudence.

Hormonal and surgical techniques for sex reassignment have advanced a lot, but they are not free of risks and undesirable side effects, especially regarding the genitals. For this reason and for the options of living fully without assuming the normative conditions of gender in force, the debate is increasingly open and the need to completely transform and, in all cases, the sexual characteristics is questioned. Metaphorically, one can question whether people who experience an inconsistency between their self-perception of gender and their body characteristics should admit that they “were born in the wrong body” or that “they must conquer the body based on their identity”^(4,7)^.

Nurses as health workers, and from their role as advocates for users of the health system, can assume several general lines of action in the face of the trans phenomenon:

Monitoring and facilitating those who carry out the transition process in their passage through the complex labyrinth of the health system.

Accompanying and advising families and the educational community with doubts about the identity of their children, out of respect, but also out of prudence.

Dissemination of scientific knowledge about the trans phenomenon in society to promote respect for sexual diversity and their rights.

For all these reasons, complementary training should be provided to professionals and students of nursing and other health sciences, with the involvement of both university centers, as well as collegiate entities and professional scientific societies.
