# ERRATUM

**DOI:** 10.1590/0034-7167.20247701e01

**Published:** 2024-01-15

In the article “The ethics of nursing care for transgender people”, with DOI number: https://doi.org/10.1590/0034-7167-2022-0797, published in Revista Brasileira de Enfermagem, 2023;76(Suppl 3):e20220797, in authorship:

Where it read:

**Enrique Oltra Rodríguez^I^**

ORCID: 0000-0002-9124-5550

**Eva González López^II^**

ORCID: 0000-0001-7653-0110

**Sofía Osorio Álvarez^I^**

ORCID: 0000-0002-0624-9259

**Andrea Rodríguez Alonso^I^**

ORCID: 0000-0002-5722-2662

**How to cite this article:**

Rodríguez EO, López EG, Álvarez SO, Alonso AR. The ethics of nursing care for transgender people. Rev Bras Enferm. 2023;76(Suppl 3):e20220797. https://doi.org/10.1590/0034-7167-2022-0797

**Corresponding author:** Enrique Oltra Rodríguez E-mail: kikeoltra@gmail.com

It reads:

**Enrique Oltra-Rodríguez^I^**

ORCID: 0000-0002-9124-5550

**Eva González-López^II^**

ORCID: 0000-0001-7653-0110

**Sofía Osorio-Álvarez^I^**

ORCID: 0000-0002-0624-9259

**Andrea Rodríguez-Alonso^I^**

ORCID: 0000-0002-5722-2662

**How to cite this article:**

Oltra-Rodríguez E, González-López E, Osorio-Álvarez S, Rodríguez-Alonso A. The ethics of nursing care for transgender people. Rev Bras Enferm. 2023;76(Suppl 3):e20220797. https://doi.org/10.1590/0034-7167-2022-0797

**Corresponding author:** Enrique Oltra-Rodríguez E-mail: kikeoltra@gmail.com

